# Predicted distribution of *Metaparasitylenchus hypothenemi* (Tylenchida: Allantonematidae), parasite of the coffee berry borer

**DOI:** 10.2478/jofnem-2024-0031

**Published:** 2024-08-06

**Authors:** M. Simota-Ruiz, A. Castillo, J. Cisneros-Hernández, O. Carmona-Castro

**Affiliations:** El Colegio de la Frontera Sur, Carretera Antiguo Aeropuerto Km. 2.5, Tapachula, CP 30700, Chiapas, México

**Keywords:** species distribution models, geographical information systems, *Hypothenemus hampei*, Coleoptera, Curculionidae

## Abstract

*Metaparasitylenchus hypothenemi* is an endoparasitic nematode of the coffee berry borer *Hypothenemus hampei*. The nematode has only been recorded across a limited geographical range in coffee-growing areas of southeastern Mexico. Because of its confined geographical distribution, the effect of altitude, temperature, and mean annual precipitation on *M. hypothenemi's* presence/absence in the Soconusco region of Mexico was investigated. The geographical distribution of this parasite was predicted based on current data, using geographical information systems (GIS), the MaxEnt algorithm, and historical data to improve the prediction accuracy for other Neotropical regions. In Soconusco, the presence of this parasite is directly related to annual precipitation, especially in the areas with the highest annual rainfall (4000 – 4700 mm/year). Four species distribution models were generated for the Neotropical region with environmental variables for sites with parasite presence data, predicting a range of possible distribution with a high probability of occurrence in southeastern Mexico and southwestern Guatemala and a low probability in areas of Central and South America. Characterization of the abiotic habitat conditions suitable for *M. hypothenemi* development allows us to predict its distribution in the Neotropics and contributes to our understanding of its ecological relationship with environmental variables.

The nematode *Metaparasitylenchus hypothenemi* Poinar (Nematoda: Allantonematidae) is an endoparasite that can induce sterility in the coffee berry borer *Hypothenemus hampei* Ferrari (Coleoptera: Curculionidae: Scolytinae) (Castillo et al., 2019), the most serious pest in coffee plantations worldwide (Le Pelley, 1968; Vega et al., 2015). *Metaparasitylenchus hypothenemi* has been observed in only a few localities in Chiapas, Mexico (Pérez et al., 2015), where it was first recorded parasitizing females of *H. hampei* (Castillo et al., 2002). Genetic studies suggest that this nematode is likely endemic to the Neotropical region and that the borer is a factitious host (unpublished data, Simota et al.). However, the ecological relationship between the parasitic nematode and the coffee berry borer or other possible hosts has remained relatively underexplored. Also, the parasite’s natural habitat and its capacity to adapt to precipitation, temperature, and relative humidity levels are still largely uncharacterized. These environmental variables are important because they affect the survival and distribution of insect-parasitic nematodes (Robbins and Barker, 1974; Grewal et al., 1994) and may provide a way to gain insight into the nematode’s narrow geographical distribution range.

The increasing development and application of geographical information systems (GIS) and species distribution models (SDM) have facilitated the study and understanding of numerous ecological and geographical phenomena of various mammalian parasitic nematodes (Haverkost et al., 2010; Martínez-Salazar et al., 2013; Feidas et al., 2014). However, no SDM studies of nematode parasites of agricultural pests are available. The present work analyzes the effect of altitude, temperature, and precipitation on the presence/absence of *M. hypothenemi* in the Soconusco, Chiapas, Mexico. Its geographical distribution was modeled using GIS and the MaxEnt algorithm (Maximum Entropy - Species Distribution Modeling) (Phillips et al., 2006) for the rest of the Neotropical region from current and historical data.

## Materials and Methods

*Sample collection:* Adult *H. hampei* populations from coffee plantations across Soconusco, Chiapas, Mexico, and from one site in San Pablo, San Marcos Department, Guatemala, were examined for parasitism by the nematode *M. hypothenemi*. Sampling occurred in 2003 and 2019 at 59 sites distributed over the study area. In 2003, adult coffee berry borers were captured in 23 coffee plantations using traps baited with a methanol/ethanol mixture (1:1 by volume), placed in each plantation, and suspended from the middle part of a coffee shrub during the rainy season (April–July 2003). In 2019, remaining robusta coffee berries were collected directly from the plant during the dry season (January–May 2019) in 36 coffee plantations, and adult berry borers were extracted from them. Sites were chosen based on various criteria: high shade density, low crop management practices, soil with high organic matter content, and high relative humidity (Perez et al., 2015). In both samples, nematode infection was determined by dissection of the captured borers under a stereo microscope.

*Influence of altitude, temperature, and precipitation on nematode presence:* Sample site altitude was recorded using a GPS device (Garmin Oregon 350; Garmin, Inc., Taipei, Taiwan). Mean annual temperature and precipitation data were obtained from the climate database WorldClim (https://www.worldclim.org/, consulted 2020). The bioclimatic variables BIO_1_ (mean annual temperature) and BIO_12_ (annual precipitation) were updated for Mexico (30 arc sec resolution, approximately 90 m / pixel) (Cuervo-Robayo et al., 2013). Data were obtained for each sampling site using the point sampling tool in the software program QGIS 3.10.3 (https://qgis.org/es/site/) (QGIS, 2018).

A multivariate analysis of variance (MANOVA) was performed to analyze the effect of altitude, annual precipitation, and temperature, with and without presence/absence data from 2003 and 2019. The missForest package was used to estimate two missing data for temperature and annual precipitation (Stekhoven and Bühlmann, 2012). A canonical discriminant analysis was performed to identify the environmental variables that were most strongly associated with nematode presence. A Chi-square test was performed using the statistical software package R (version X) to determine whether the presence of *M. hypothenemi* varied across the four different annual rainfall distribution ranges of the Soconusco (R Core Team, 2019).

*Species distribution modeling (SDM):* A model for the geographical distribution of *M. hypothenemi* was calibrated for the Neotropical Biogeographical Region, which includes all the coffee-growing regions of the Americas. Unpublished nematode parasitism data from 2003 were incorporated to improve model accuracy. A species distribution model (SDM) was generated with the MaxEnt (Maximum Entropy-Species Distribution Modeling, Version 3.4.1) algorithm (Phillips et al., 2006), using data from 29 sites where the parasite was present, climate variables from WorldClim, and Shuttle Radar Topography Mission (SRTM) altitude data from the US Geological Survey (USGS, http://www.usgs.gov) with a resolution of 30 arc sec (~1 km_2_). MaxEnt was used under linear constraints, with random selection of 30% of the presence sites, minimum training presence, and 500 iterations (Anderson et al., 2003). The first model (model A) was generated using 15 climate variables – BIO_1_ (annual mean temperature), BIO_2_ (mean diurnal range), BIO_3_ (isothermality), BIO_4_ (temperature seasonality), BIO_5_ (max temperature of warmest month), BIO_6_ (min temperature of coldest month), BIO_7_ (temperature annual range), BIO_10_ (mean temperature of warmest quarter), BIO_11_ (mean temperature of coldest quarter), BIO_12_ (annual precipitation), BIO_13_ (precipitation of wettest month), BIO_14_ (precipitation of driest month), BIO_15_ (precipitation seasonality), BIO_16_ (precipitation of wettest quarter), BIO_17_ (precipitation of driest quarter) – and the altitude, without considering a possible correlation between the variables. The climate variables, BIO_8_ (mean temperature of wettest quarter), BIO_9_ (mean temperature of driest quarter), BIO_18_ (precipitation of warmest quarter), and BIO_19_ (precipitation of coldest quarter), were not included because of odd discontinuities between neighboring pixels (Escobar et al. 2014). The second model (model B) was constructed using eight climate variables (BIO_1_, BIO_2_, BIO_3_, BIO_7_, BIO_12_, BIO_13_, BIO_15_, and BIO_16_) and the altitude, which were selected after multicollinearity analysis because of their low correlation (r < 0.75). The third model (model C) included the six climate variables with the highest percentage of contribution according to the Jackknife test (> 0.95; BIO_2_, BIO_3_, BIO_12_, BIO_13_, BIO_15_, BIO_16_, and the altitude). To improve prediction accuracy, the summation of these three models was used to generate the final model (model D). Model precision was evaluated using partial ROC analysis, and the errors of omission and predicted proportional areas were considered satisfactory (error of omission (E) = 5%, 50% data for evaluation, bootstrap=1000) (Escobar et al., 2013). The models were evaluated with the PartialROC function (Barve, 2008), and the geographical accuracy of the model was assessed by projecting presence and absence data onto the final SDM (model D). A threshold was selected to include at least 90% of the presence data (27 records), which was projected onto the categories with the highest probability of occurrence (> 6) for the transformation of SDM to the binary model (presence = 1/absence = 0). The binary models were used to estimate the breadth of each SDM (1 pixel = 1 km^2^) (Moo-Llanes et al., 2013).

## Results

**Figure 1.**
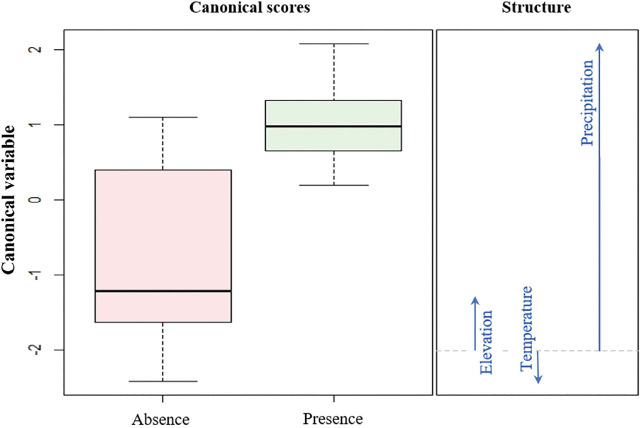
Canonical discriminant analysis based on environmental variables (altitude, temperature, and precipitation) to model the presence/absence of the nematode *Metaparasitylenchus hypothenemi* in the Soconusco, Chiapas, for the year 2003. Variability explained by the canonical variable is 100%.

**Figure 2.**
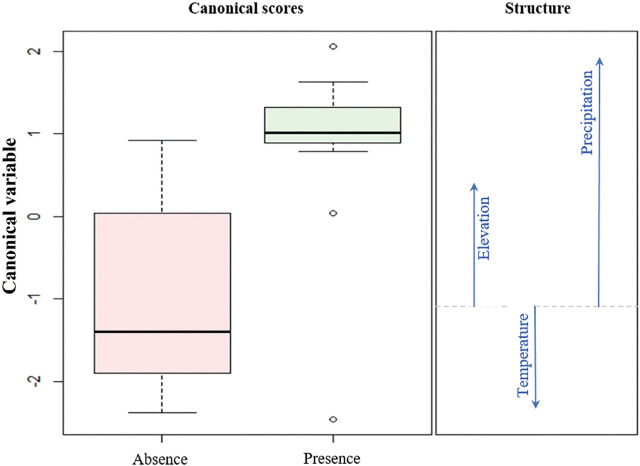
Canonical discriminant analysis based on environmental variables (altitude, temperature, and precipitation) to model the presence/absence of the nematode *Metaparasitylenchus hypothenemi* in the Soconusco, Chiapas, for the year 2019. The variability explained by the canonical variable is 100%.

*Influence of altitude, temperature, and precipitation on nematode presence:* Parasitism was detected in 29 out of 59 investigated sites (Tables 1, 2). Multivariate analysis of variance (MANOVA) showed significant differences in the binomial response (presence-absence) to environmental variables (altitude, precipitation, and temperature) in data from 2003 (F = 6.1; g.l. = 3; p = 0.003), as well as 2019 (*F* = 10.5; g.l. = 3; *P* < 0.001). Canonical discriminant analysis revealed that in both years, the presence of *M. hypothenemi* was associated with precipitation, while its absence was associated with temperature and altitude (Figs. 1, 2).

**Table 1.** Altitude, temperature, and precipitation for 2003 and 2019 in coffee plantations with *Metaparasitylenchus hypothenemi* presence (various localities in Mexico and San Pablo, Guatemala).

**Year**	**Municipality**	**Locality**	**Altitude (m a.s.l.)**	**Temperature (°C)**	**Precipitation (mm/year)**
2003	Unión Juárez	Finca Monte Perla	1020	22.87	4311.96
		Ejido Once de Abril	852	23.50	4544.00
	Cacahoatán	Ejido Santo Domingo	810	23.79	4640.05
		Finca La Alianza	671	24.24	4503.66
		Finca El Zapote	676	24.21	4525.80
		Rancho El Paraíso	564	24.65	4433.28
	Tapachula	Finca San Enrique	754	23.99	4139.28
		Ejido Cinco de Mayo	554	24.85	4315.99
		Ejido El Edén	544	25.57	3875.75
		Rancho La Esperanza	507	25.14	4134.87
	Tuxtla Chico	Finca El Encanto	461	25.03	4242.72

2019	Unión Juárez	Río Suchiate	1014	22.00	3295.00
		Finca Monte Perla	938	23.18	4443.20
		Santo Domingo	883	23.47	4636.51
		Ejido Once de Abril	850	23.56	4562.55
		San Rafael	819	23.63	4629.64
		Ejido San Jerónimo	747	23.94	4573.30
	Cacahoatán	Dos de Mayo	839	23.64	4566.13
		Faja de Oro	836	23.60	4559.70
		Finca El Zapote	720	24.09	4547.48
		Finca La Alianza	703	23.92	4563.86
		Rosario Ixtal	600	24.59	4455.24
		Rancho San Antonio	577	24.61	4447.98
		La Unidad	568	24.63	4443.94
	Tapachula	Finca Santa Lucía	672	24.26	4426.17
		Salvador Urbina	598	24.59	4439.59
		Finca Brasil	463	25.31	4045.24
	Acacoyagua	Ejido Los Cacaos	450	25.25	3211.97
	San Pablo	Finca Buena Vista	670	24.00	3689.00

**Table 2.** Altitude, temperature, and precipitation for 2003 and 2019 in coffee plantations with *Metaparasitylenchus hypothenemi* absence.

**Year**	**Municipality**	**Locality**	**Altitude (m a.s.l.)**	**Temperature (°C)**	**Precipitacion (mm/year)**
2003	Unión Juárez	Unión Juárez	1295.00	21.51	4013.41
	Cacahoatán	Finca La Gloria	569.00	24.64	4436.73
	Tuxtla Chico	Ejido Manuel Lazos	363.00	25.45	3834.87
		San José La Victoria	484.00	24.96	4286.24
	Tapachula	Finca Hamburgo	1166.00	22.18	3729.59
		Finca Irlanda	1149.00	22.32	3694.39
		Finca Maravillas	647.00	24.28	4290.40
		San Miguel	409.00	25.45	3943.71
	Huixtla	Camino Real	465.00	25.16	3631.59
		Finca Córcega	370.00	25.23	3628.14
	Villacomaltitlán	Ejido Zapote Mocho	120.00	26.85	3329.28
		Finca La Granja	459.00	24.87	3638.88

2019	Motozintla	Finca Santa Catalina	795.00	23.52	3363.59
		Ranchería Varitas	757.00	23.67	3376.80
		El Manguito	748.00	23.87	3464.51
		Finca Brasil	734.00	23.80	3441.29
		Los Cocos	637.00	24.34	3642.55
	Tapachula	Ejido Mexiquito	813.00	23.63	4072.84
		Santa Anita	766.00	23.76	4041.23
		Finca Maravillas	760.00	23.83	4148.71
		Tiro Seguro	713.00	24.08	4130.85
		San José Nexapa	665.00	24.30	4429.00
		Ejido Toluca	553.00	24.76	4372.55
		Malpaso	326.00	25.85	3554.65
	Tuxtla Chico	Finca El Encanto	461	25.14	4176.88
	Escuintla	Ejido Tres de mayo	529.00	25.06	3377.83
		Jamaica	331.00	25.99	3441.58
	Tuzantán	El Retiro	480.00	25.22	3818.76
		Río Negro	208.00	26.65	3339.10
	Villacomaltitlán	Manuel Ávila Camacho	109.00	27.05	3249.11

**Figure 3.**
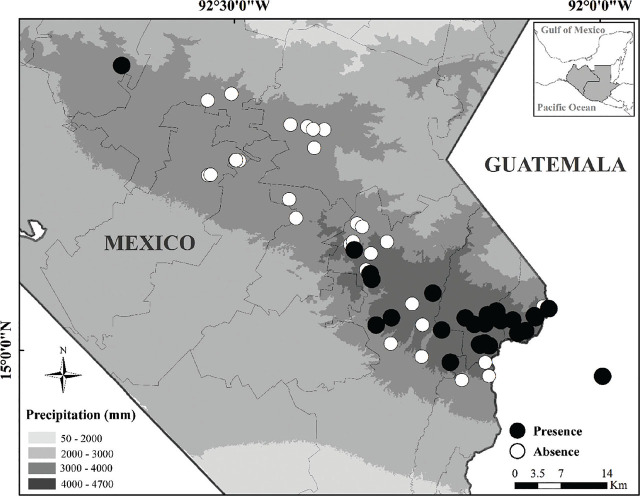
Distribution of sites with presence of the nematode *Metaparasitylenchus hypothenemi* across four different rainfall distribution ranges of the Soconusco for the years 2003 and 2019. Site locations appear as white (absence) or black (presence) dots.

Sites with nematode presence were located between 450 and 1020 masl, in areas where the annual mean temperature ranges between 22°C and 25.6°C. However, 86% of the sites with nematode presence were located in the area with the highest annual rainfall (4000–4700 mm), so the probability of locating sites with nematode presence is lower in areas where annual rainfall is less than 4000 mm (χ^2^ = 73.4; g.l. = 4; *P* < 0.001) (Fig. 3). Because 10 out of 59 sampled sites were in the same locality, only 54 points are marked on the map. The predictive performance of the models was significant (partialROC > 1; Table 3) with a similar amplitude and geographical distribution in all four models.

**Table 3.** Breadth and evaluation of the SDMs. All models were characterized by a high predictive accuracy in the partial ROC test (AUC = 0.99). The breadth of the geographical distribution of *Metaparasitylenchus hypothenemi* was similar (4692 km^2^), although all models predict a limited geographical distribution (0.02%) for the Neotropical region (23943229 km^2^).

**SDM**	**Accessible area**	**SDM breadth**	**Proportion**	**Partial ROC test**		

	**(1px = 1km^2^)**	**(1px = 1km^2^)**	**(%)**	**AUC of 0.95**	**AUC of 0.5**	**Ratio**
Model A	23,943,229	4,977	0.021	0.99976	0.49999	1.99951
Model B		4,811	0.020	0.99979	0.49999	1.99959
Model C		4,834	0.020	0.99980	0.49999	1.99959
Final model (D)		4,692	0.020	-	-	-

**Figure 4.**
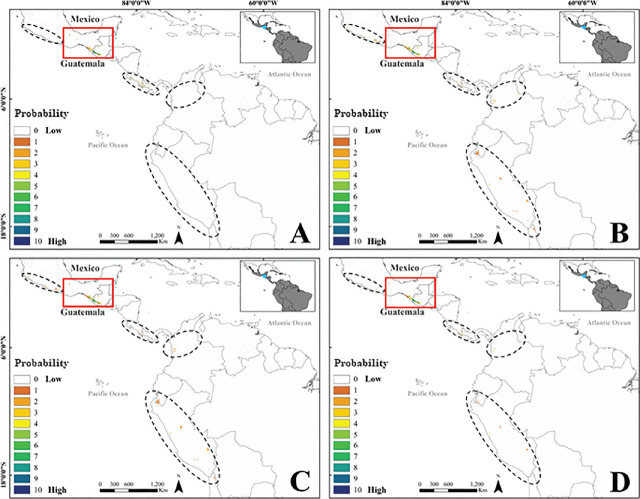
Models of geographical distribution of *Metaparasitylenchus hypothenemi* in the Neotropical region. A) with fifteen climatic variables, B) with eight climatic variables, C) with six climatic variables, and D) the final model constructed with the sum of models A, B, and C. Regions with probability of occurrence (high inside the red rectangle, low inside the dotted line shapes).

**Figure 5.**
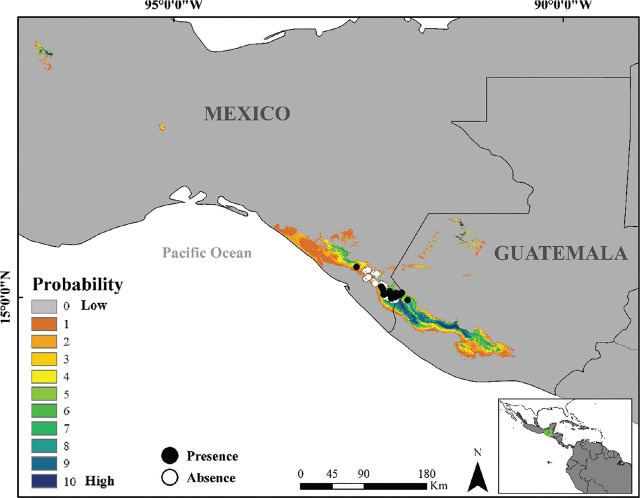
Zoomed-in on the region with the highest probability of occurrence (Mexico and Guatemala), with dots indicating site locations with absence (white) and presence (black) of the parasite for the years 2003 and 2019. The probability of nematode occurrence is shown as a color gradient (1–10).

The final SDM model is shown in Fig. 4D. All models predicted areas with low probability of nematode presence in western Mexico, Central America (Costa Rica and Panama), and South America (Colombia, Ecuador, and Peru). The presence of parasitism appeared in areas of high probability of occurrence (86% in categories > 6), whereas its absence fell in areas of low occurrence probability (65% in categories < 4) (Fig. 5). The average SDM extent was 4692 km^2^. Areas with high probability of occurrence were concentrated in southeastern Mexico and Guatemala (Fig. 5).

## Discussion

Our results demonstrate that natural populations of this nematode are adapted to the environmental conditions of Soconusco and have persisted for at least the last 20 years (Castillo et al., 2002; Poinar et al., 2004; Pérez et al., 2015). Precipitation, in particular, affects the presence of this parasite in the study region, whereas its absence is associated with altitude and temperature. Although no parasitism data are available for the period 2004–2018, our results indicate a direct relationship between the presence of this species and rainfall. Moreover, since the duration of the rainy season in the study region is six to ten months, our results also suggest temporal patterns of parasitism. The dynamics of these patterns, however, have remained a relatively ill-explored research topic, and further research in this field is still necessary. Sites with the highest probability of nematode occurrence fall in the areas with the highest annual precipitation (> 4000 mm), between 450 and 1020 masl. At these altitudes, the dry season lasts only two months (December–January), which reduces the risk of dehydration and allows the nematodes to maintain a permanent presence. The coffee berry borer depends on humidity for its survival during the critical phases of its life cycle, including emergence from the fruit, as well as colonization, penetration, and development inside the fruit (Baker et al., 1994). Borer populations accumulate in residual coffee berries, ensuring their survival and development during the dry season (Damon, 2000). These factors might also favor transmission and persistence of the parasite since nematodes are highly susceptible to dehydration and sunlight (Banu and Rajendran, 2003). However, it remains unknown whether *H. hampei* is the only natural host of *M. hypothememi*. The coffee berry borer is an exotic pest that invaded Mexican coffee plantations in 1978 (Baker, 1984), whereas parasite populations might be endemic (unpublished data, Simota et al.).

The model of the possible distribution of *M. hypothenemi* in the Neotropics predicted with a high degree of probability the presence of this nematode in southeastern Mexico and southwestern Guatemala. These regions include high-altitude and humid areas with particular abiotic conditions that allow for high levels of endemism (Morrone and Márquez, 2003). The present study is the first published investigation that applies this type of model to evaluate the geographical distribution of an insect-parasitizing nematode of agro-economic importance. However, its anecdotal presence in Honduras (Poinar et al., 2004) and present observation in Guatemala suggest that, as predicted by the model, nematode populations are distributed in plantations in Central America.

Species distribution is geographically constrained by environmental conditions and is ultimately determined by climatic tolerances and evolutionary processes (Wiens and Graham, 2005). Environmental and geographical space models were generated for the Neotropical region, which includes various large coffee-growing regions. The coffee berry borer is an important pest in these regions and is now known to complete its life cycle only in coffee berries (Johnson et al., 2020). The model predicts small areas in South America where *M. hypothenemi* can be encountered. However, because the abiotic conditions that are essential for the survival of this parasite are not adequately matched, the probability of occurrence in these regions is considered low. Finding other areas where the parasite is present would allow confirmation of the model’s predictions and, as such, continues to be a priority. The model is the first theoretical tool available, and its predictive validity is evidenced by the suitable projection of data from areas with high and especially low probabilities of occurrence, allowing us to predict the possible geographical distribution of *M. hypothenemi* with a high degree of reliability.

Although climate change is only one of the many factors that contribute to the decline of a species’ population, it has been identified as one of the main causes of species extinction (Román-Palacios and Wiens, 2020). The models presented here indicate that the presence and distribution of *M. hypothenemi* may be affected by climate change because the parasite is geographically restricted to areas of high precipitation rates in southeastern Mexico and Central America, and its potential distribution in other regions of the Neotropics is limited. Even though extinction has not been documented for any species of parasitic nematode, endemic species are usually more vulnerable to climate change (Carlson et al., 2017), which highlights the need to design conservation plans for this species.

The parasite *M. hypothenemi* plays a role in the natural regulation of coffee berry borer populations by significantly reducing the fecundity of infected hosts (Poinar et al., 2004; Castillo et al., 2019). Although there is no direct evidence that the parasite causes the mortality of its host, parasite-induced sterility in females of the coffee berry borer may lead to a population decrease in the following generations of this pest (Castillo et al., 2002; Castillo et al., 2019). Further research is needed to fully understand the bioecology of this parasite to chart its demography and explore host-parasite interactions under controlled conditions. Likewise, additional studies focusing on the parasite’s genetic diversity can shed light on its geographical origin and hence provide valuable input to establish priority conservation areas. Prediction models can be a useful tool to identify sites in other countries of the Americas where *M. hypothenemi* may be present and contribute to the understanding of the ecological relationships between the parasite and the environment.
